# Synergistic Ion-Releasing Nanoparticles as a Therapeutic Platform for Modulating Adult Stem Cell Activity in Wound Healing

**DOI:** 10.34133/bmr.0281

**Published:** 2025-12-03

**Authors:** Yu-Jin Kim, Jaeyoung Lee, Eun-Cheol Lee, Jiwoo Song, Yonghwan Jo, Han Young Kim, Taekyung Yu, Suk Ho Bhang

**Affiliations:** ^1^ School of Chemical Engineering, Sungkyunkwan University, Suwon 16419, Republic of Korea.; ^2^ Biomaterials Research Center, Korea Institute of Science and Technology, Seoul 02792, Republic of Korea.; ^3^ BK21 FOUR Integrated Engineering Program, Department of Chemical Engineering, Kyung Hee University, Yongin 17104, Republic of Korea.; ^4^ School of Earth and Atmospheric Sciences, Georgia Institute of Technology, Atlanta, GA 30332, USA.; ^5^ Department of Biomedical-Chemical Engineering, The Catholic University of Korea, Bucheon 14662, Gyeonggi, Republic of Korea.

## Abstract

Nanoparticles are increasingly utilized for their potential in targeted drug delivery, highlighting the need for innovative approaches to enhance therapeutic and regenerative outcomes. This study investigated zinc- and iron-ion-releasing nanoparticles (ZFNs) for their ability to simultaneously deliver zinc (Zn) and iron (Fe) ions, aimed at boosting the efficacy of human mesenchymal stem cells (hMSCs) in wound healing. Engineered for pH-sensitive degradation, ZFNs enable the controlled intracellular release of these ions following endocytosis by hMSCs. Our in vitro findings include favorable release kinetics and the absence of toxicity. We observed that dual-ion delivery via ZFNs markedly modulated the key zinc transporter gene expression and enhanced the angiogenesis- and migration-related gene expression in hMSCs. This activity correlates with the activation of mitogen-activated protein kinase and AKT signaling pathways, essential for processes such as cell migration and proliferation, thereby supporting tissue regeneration. Indeed, changes in the secretion profiles of hMSCs treated with ZFNs were found to enhance the migratory and regenerative capacities of both fibroblasts and keratinocytes. In vivo experiments confirmed that hMSCs integrated with ZFNs accelerate wound healing and upregulate the expression of essential skin barrier proteins. Collectively, these findings position ZFNs as a promising tool for enhancing stem-cell-mediated tissue regeneration, with potential widespread applications in clinical stem cell therapies.

## Introduction

Human mesenchymal stem cells (hMSCs) are essential in wound repair for their ability to emit growth factors that enhance tissue regeneration by promoting cell proliferation, angiogenesis, and extracellular matrix deposition [1]. Nonetheless, the effectiveness of stem cell therapy hinges on the local microenvironment and the precise release of these bioactive substances [2]. To enhance the regenerative capabilities of hMSCs, recent advances have focused on the application of nanoparticles (NPs) to modulate the cellular microenvironment. Among these advancements, NPs like cerium oxide, copper oxide, and iron oxide have shown potential in releasing therapeutic ions directly within cells [3–5]. An intriguing development in this area involves NPs that are specifically designed to respond to the acidic pH of endosomes, a common environment encountered once NPs are internalized by cells via endocytosis. This endosome-specific pH responsiveness allows for the controlled release of ions precisely where they can exert marked therapeutic effects, enhancing the regenerative potential of stem cells [6]. Wound healing is a complex biological process that includes a cascade of cellular and molecular events aimed at restoring damaged tissue integrity, encompassing inflammation, tissue formation, and remodeling phases—each critical for successful recovery [7]. Within this intricate mechanism, stem cell therapy has emerged as a promising strategy, enhancing healing through cellular regeneration and functional restoration, where a tailored release mechanism of therapeutic agents is especially critical for promoting effective tissue repair and regeneration [7].

In recent years, zinc- and iron-ion-releasing NPs (ZnFe_2_O_4_, ZFNs) have gained considerable attention in wound healing due to their remarkable antimicrobial properties and ability to promote tissue regeneration [8,9]. These NPs effectively combat drug-resistant bacterial infections through reactive oxygen species (ROS) generation and support cell migration and proliferation, making them a promising candidate for advanced wound care applications. Additionally, ZFNs exhibit excellent biocompatibility, showing minimal cytotoxicity and hemocompatibility, further reinforcing their potential as a novel therapeutic agent in dermatological treatments [8,9]. The simultaneous delivery of iron and zinc ions from ZFNs is particularly advantageous for wound healing as it synergistically supports cellular processes involved in tissue regeneration, with iron supporting angiogenesis and zinc enhancing cell migration. Our previous studies have demonstrated that iron ions delivered through NPs can enhance stem cells’ angiogenesis and migration ability, thereby improving wound healing outcomes [10]. Additionally, zinc ions promote the adhesion, migration, and self-renewal capacity of stem cells [11]. However, to our knowledge, the specific effects of dual-ion delivery using ZFNs on stem cells have not been thoroughly investigated. Our study aims to address this gap, potentially contributing to the development of novel therapeutic strategies in regenerative medicine and targeted wound healing.

The utilization of ZFNs for dual-ion release introduces a novel method for enhancing the intracellular ion concentration in a controlled manner. By dissolving into ions within the cell, these NPs can deliver iron and zinc ions directly to the intracellular space, effectively avoiding prolonged biopersistence and potential cytotoxicity [12]. These ions play a pivotal role in cellular functions, as they are essential components in numerous enzymatic processes and are critical for maintaining ion homeostasis. Various proteins and ion transporters within the cells work tirelessly to sustain this balance, adjusting to environmental changes to preserve cellular functionality [13,14]. This dual-ion delivery mechanism underscores the importance of ZFNs in stem cell therapy, as the combined effect of iron and zinc ions markedly improves the overall healing efficacy by promoting a more robust regenerative response in hMSCs.

In this study, we explored the multifaceted role of ZFNs in enhancing stem-cell-mediated wound healing. We specifically focused on their pH-responsive ion-releasing capability, the consequent improvement in wound healing efficacy, and the stimulation of skin barrier protein regeneration. We also examined the underlying mechanisms by which zinc and iron ions contribute to cellular functions and how their controlled release can be harnessed to maximize the therapeutic potential of stem cells. By integrating these NPs into stem cell therapy, we aim to establish a novel and effective approach for enhancing wound healing and tissue regeneration, promising a marked stride forward in regenerative medicine and therapeutic interventions.

## Materials and Methods

### Experimental and technical design

This study was designed to evaluate whether ZFNs could enhance stem-cell-mediated wound healing through controlled dual-ion release. We synthesized ZFNs and characterized their pH-responsive ion release. hMSCs were treated with ZFNs to examine cellular responses, including viability, ROS generation, and gene expression. We then assessed how these changes influenced fibroblast and keratinocyte behavior through paracrine signaling. Finally, the therapeutic potential of ZFN-integrated hMSCs was evaluated in a murine full-thickness wound model, confirming their regenerative efficacy in vivo.

### Materials

Zinc(II) chloride hexahydrate (98%, Zn(NO_3_)_2_·6H_2_O), iron(II) chloride (98%, FeCl_2_), polyethylene glycol (PEG, molecular weight = 20,000), sodium hydroxide (97%, NaOH), ammonium hydroxide solution (28.0% to 30.0%, NH_4_OH), and hydrochloric acid (37%, HCl) were purchased from Sigma-Aldrich (St. Louis, MO, USA). The analytically pure chemical reagents involved in this experiment were used without further purification.

### NP synthesis

This synthesis process was based on the previous method for synthesizing ZFNs [15]; 1 mmol of Zn(NO_3_)_2_·6H_2_O, 1 mmol of FeCl_2_, 0.4 g of PEG, and 1.2 mmol of NaOH were uniformly dispersed in 2, 2, 5, and 1 ml of deionized (DI) water, respectively, to avoid local concentration differences. After mixing Zn(NO_3_)_2_·6H_2_O, FeCl_2_, and PEG solutions, the reacting solution was stirred at room temperature (RT) for 10 min. The aqueous NaOH solution was then injected, and the resulting solution was heated at 90 °C for 16 h at a stirring rate of 1,000 rpm). After the reaction, 0.2 ml of HCl solution was added to remove unreacted residues. The product was washed with DI water and centrifuged 3 times (11,000 rpm, 10 min). After the final centrifugation, the synthesized NPs were redispersed in 10 ml of DI water for future use. The ZnO NPs used as a control group were synthesized using the same procedure as that for ZFNs, except for the initial addition of FeCl_2_ and the final addition of HCl. The synthesis of iron oxide NPs followed a slightly different protocol: 4 mmol of FeCl_2_ was dispersed in 5 ml of DI water, after which 5 ml of NH_4_OH solution was added. The mixture was stirred at RT for 10 min and subsequently reacted in an autoclave at 134 °C for 3 h. The postsynthesis washing and redispersion steps were identical to those used for the other 2 types of NPs.

### Characterization of NPs

The morphology and elemental composition of NPs were analyzed using transmission electron microscopy (JEM-2100 F, Japan) and an energy-dispersive x-ray detector, respectively. Powder x-ray diffraction (XRD) patterns were measured at RT using a Rigaku D/MAX-2200PC x-ray diffractometer with graphite-monochromatized Cu Kα radiation at a 1.54-Å wavelength. X-ray photoelectron spectroscopy was conducted using PHI 5000 Versa Probe (ULVAC PHI, Osaka, Japan) and K-Alpha Spectroscopy. Inductively coupled plasma optical emission spectroscopy analyses were performed using Direct Reading Echelle ICP (Leeman, OH, USA).

### Ion release kinetics of NPs

The dispersion of ZFNs (10 ml) was partially taken, and only 1 ml of the dispersion was used for the ion release experiment. Buffer solutions with pH 4 and 7 were prepared by adjusting with HCl and NaOH, respectively. The extracted ZFN dispersion (ml) was then mixed with 4 ml of each buffer solution. Subsequently, 1.5 ml of the supernatant was collected at predetermined intervals of 4 and 24 h for ion leaching analysis.

### Cell culture

hMSCs and human dermal fibroblasts were purchased (Lonza, Walkersville, MD, USA) and cultured using Dulbecco’s modified Eagle’s medium (Gibco BRL, Gaithersburg, MD, USA) supplemented with 10% (v/v) fetal bovine serum (Gibco BRL) and 1% (v/v) penicillin/streptomycin (PS; Gibco BRL). The cell medium was changed every other day in a 5% CO_2_ incubator at 37 °C. Five to 7 passages were used for the experiments. Normal human epidermal keratinocytes were purchased from PromoCell (Heidelberg, Germany) and cultured in keratinocyte growth medium 2 (PromoCell, Heidelberg, Germany) supplemented with 1% (v/v) PS. These cells were incubated at 37 °C in 5% CO_2_. Culture media were changed every 2 d. Four to 6 passaged cells were used for the experiments.

### Cell viability

hMSC viability was measured by Cell Counting Kit-8 (Dojindo Molecular Technologies, Rockville, MD, USA) according to the manual’s instructions. hMSCs were seeded into 24-well plates (2 × 10^4^ cells/well) and then treated with various concentrations of ZFNs for 24 h. After the initial culture, the fresh medium was replaced with Cell Counting Kit-8 and the cells were incubated at 37 °C for 2 h. Finally, the absorbance was measured at 450 nm using a plate reader (Infinite F50, Tecan, Männedorf, Switzerland). Live/dead staining was performed using fluorescein diacetate (FDA, Sigma-Aldrich)/ethidium bromide (EB, Sigma-Aldrich). FDA stains the cytoplasm of viable cells; EB stains the nuclei of dead cells. The working solution was freshly prepared by mixing 10 μl of FDA stock solution (5 mg/ml in acetone) and 10 ml of EB stock solution (10 μg/ml in phosphate-buffered saline [PBS; Gibco]). The cells were incubated in FDA/EB solution at 37 °C for 5 min. Finally, they were washed using PBS and analyzed under a fluorescence microscope (DFC 3000 G, Leica, Wetzlar, Germany). The excitation wavelengths of FDA and EB are 494 and 301 nm, while the emission wavelengths are 521 and 603 nm, respectively.

### Quantitative real-time polymerase chain reaction analysis

The expression of various genes such as housekeeping (*GAPDH*), apoptosis (*CASPASE-3*), angiogenesis (*VEGF* and *EGFR*), migration (*MMP-8* and *MMP-9*), anti-apoptosis (*NF-κB*), zinc transporter (*ZIP4*, *ZIP7*, *ZIP8*, *ZIP10*, *ZIP13*, and *ZIP14*), metallothionein (*MT1A* and *MT2A*), proliferation (*KI67*), growth factor (*FGF2*), fibroblast marker (*CD44*), and keratinocyte marker (*TP63* and *KRT15*) were analyzed with quantitative real-time polymerase chain reaction (qRT-PCR). The RNA was extracted using TRIzol (Life Technologies, Inc., CA, USA) and chloroform. The samples were centrifuged for 10 min at 12,000 rpm at 4 °C. The RNA pellets were washed with 75% (v/v) ethanol in water. After drying, the samples were dissolved in RNase-free water. For qRT-PCR analysis, SsoAdvanced Universal SYBR Green Supermix (Bio-Rad, Hercules, CA, USA) and the CFX Connect RT-PCR detection system (Bio-Rad) were used. The primer sequences used for qRT-PCR (in vitro) are listed in Table 1.

**Table 1.** Sequences of qRT-PCR primers (in vitro)

Primer	Sequences
Human *GAPDH*	F: 5′-GTC GGA GTC AAC GGA TTT GG-3′ R: 5′-GGG TGG AAT CAA TTG GAA CAT-3′
Human *CASPASE-3*	F: 5′-CCT GGT TAT TAT TCT TGG CGA AA-3′ R: 5′-GCA CAA AGC GAC TGG ATG AA-3′
Human *VEGF*	F: 5′-GAG GGC AGA ATC ATC ACG AAG T-3′ R: 5′-CAC CAG GGT CTC GAT TGG AT-3′
Human *EGFR*	F: 5′-AAC ACC CTG GTC TGG AAG TAC G-3′ R: 5′-TCG TTG GAC AGC CTT CAA GAC C-3′
Human *MMP-8*	F: 5′-CAA CCT ACT GGA CCA AGC ACA C-3′ R: 5′-TGT AGC TGA GGA TGC CTT CTC C-3′
Human *MMP-9*	F: 5′-CCA CTG CTG GCC CTT CTA CG-3′ R: 5′-CGA TGG CGT CGA AGA TGT TCA C-3′
Human *NF-κB*	F: 5′-TCC GTT ATG TAT GTG AAG GC-3′ R: 5′-TTT GCT GGT CCC ACA TAG TTG C-3′
Human *ZIP4*	F: 5′-GAG CTG GGG CTG CTT CTG-3′ R: 5′-CCT CCA CAG ACA GGC ACT TT-3′
Human *ZIP7*	F: 5′-GAC CAC AAT GAC TGT CCT GCT AC-3′ R: 5′-GCT GTC AGT AGT TGC AGA CGC A-3′
Human *ZIP8*	F: 5′-CCC CAC GAG TTA GGA GAC TTT-3′ R: 5′-GCT AGC CCA ACA TAG CAG GA-3′
Human *ZIP10*	F: 5′-TTT CAC TCA CAT AAC CAC CAG C-3′ R: 5′-GTG ATG ACG TAG GCG GTG ATT-3′
Human *ZIP13*	F: 5′-TCC CCC AAG GGA GTA GTT GG-3′ R: 5′-AGG CGA TGT AGA GAA AGC CG-3′
Human *ZIP14*	F: 5′-GCT TAT GGA GAA CCA CCC CT-3′ R: 5′-AGG TTC CTG TGT CCT TGC AC-3′
Human *MT1A*	F: 5′-AGA GTG CAA ATG CAC CTC CTG C-3′ R: 5′-CGG ACA TCA GGC ACA GCA GCT-3′
Human *MT2A*	F: 5′-CAA CTG CTC CTG CGC CG-3′ R: 5′-CAG CAG CTG CAC TTG TCC G-3′
Human *KI67*	F: 5′-TGA CCC TGA TGA GAA AGC TCA A-3′ R: 5′-CCC TGA GCA ACA CTG TCT TTT-3′
Human *FGF2*	F: 5′-AGC GGC TGT ACT GCA AAA AC-3′ R: 5′-GTA GCT TGA TGT GAG GGT CG-3′
Human *TP63*	F: 5′-GAT TCA TAT TGT AAG GGT CTC GG-3′ R: 5′-GGG CAT TGT TTT CCA GGT A-3′

qRT-PCR, quantitative real-time polymerase chain reaction

### Measurement of the metal ion concentration

The elemental analysis of the ZFNs was performed using an inductively coupled plasma atomic emission spectroscopy (ICP-AES) spectrometer (Direct Reading Echelle ICP, Leeman, OH, USA). To analyze the NP cellular uptake using ICP-AES, hMSCs were seeded into 6-well plates (1 × 10^5^ cells/well) and incubated for 24 h with ZFNs. Cells were washed with PBS and dissolved with nitric acid hydrochloride (a mixture of nitric acid and hydrochloric acid in a molar ratio of 1:3) overnight. Ionized samples were diluted in DI water (1:4 [v/v]). The iron and zinc concentration were determined using ICP-AES.

### Intracellular distribution of NPs

To observe the intracellular localization of ZFNs, hMSCs were seeded into 150-mm culture dishes and treated with ZFNs (5 μg/ml) for 24 h. After treatment, the cells were fixed overnight at 4 °C using Karnovsky’s fixative and rinsed 3 times with 0.05 M sodium cacodylate buffer. Postfixation was performed with 1% osmium tetroxide for 1 h at RT, followed by triple washing with distilled water. The samples were then stained with 0.5% uranyl acetate overnight at 4 °C and dehydrated through a graded ethanol series (30%, 50%, 70%, 80%, 90%, and 100%). After dehydration, the specimens were embedded in Spurr’s resin and polymerized at 70 °C for 24 h. Ultrathin sections (approximately 100 nm thick) were prepared using an ultramicrotome (Lecia, Wetzlar, Germany), mounted on 200-mesh copper grids and examined using a transmission electron microscope (JEM-1010, JEOL, Tokyo, Japan).

### Analysis of hydroxyl radical (·OH) generation

To assess the ·OH generation capability of ZFNs, a methylene blue (MB; Sigma-Aldrich) degradation assay was conducted. ZFNs (30 μg/ml) were incubated under different pH conditions (pH 4 and pH 7) for 24 h to induce ion release. Following incubation, MB (100 μM) and H_2_O_2_ (10 μM) were added to the samples, and the mixtures were incubated at 37 °C for 1 h. The absorbance at 664 nm was measured using Cary 60 ultraviolet–visible spectroscopy (Agilent Technologies, CA, USA). A decrease in MB absorbance indicated ·OH production through Fenton-like reactions.

### Measurement of intracellular ROS levels

After 24 h of treatment, intracellular ROS levels were measured using 2,7-dichlorodihydrofluorescein diacetate (DCFDA; Invitrogen, Carlsbad, CA, USA). Briefly, SC or SC@ZFNs were stained with 5 μM DCFDA using 1% PS medium at 37 °C. The intracellular generated ROS level was analyzed using a fluorescence microscope (DFC 3000 G, Leica, Wetzlar, Germany) and fluorescence-activated cell sorting (FACS; MACSQuant VYB, Miltenyi Biotec) after the cells were washed twice with PBS and FACS buffer solution, respectively. Fluorescent signals were quantified using the FlowJo software (Becton, Dickinson and Company).

### Western blot analysis

The SC or SC@ZFNs were lysed using radioimmunoprecipitation assay buffer (Rockland Immunochemicals Inc., Limerick, PA, USA), and a protease and phosphatase inhibitor cocktail was added. The protein concentration was determined with a bicinchoninic acid protein assay (Pierce Biotechnology, Rockford, IL, USA). Western blot analysis was performed by using the protocols reported previously [16]. The blots were developed in a dark room using a luminescence-recording x-ray film (blue color; Agfa HealthCare NV, Mortsel, Belgium).

### Paracrine influence of SC@ZFNs on fibroblasts and keratinocytes

To evaluate the paracrine effects of SC@ZFNs on fibroblasts and keratinocytes, a transwell co-culture system (0.4-μm pore size, Corning, NY, USA) was used [17]. Fibroblasts and keratinocytes were seeded into the bottom chamber of 6-well plates at full confluency, while SC or SC@ZFNs were seeded into the upper transwell inserts. The bottom-layer cells were subjected to the wound healing assay. A straight scratch was made on the plates using an SPLScar Scratcher (SPL Life Sciences, Pocheon, Korea). After 24 h of co-culture, the relative gap area was calculated compared to that at 0 h [18]:

$$\begin{array}{c} \begin{array}{c} \text{Scratch}\;\text{closure}\left(\%\right)=\\ \left[\left(\text{initial}\;\text{scratch}\;\text{area}-\text{scratch}\;\text{area}\;\text{at}24\;\text{h}\right),/\text{initial}\;\text{scratch}\;\text{area}\right]\times 100 \end{array} \end{array}$$ (1)

To assess the gene expression changes, total RNA was extracted from fibroblasts and keratinocytes after 24 h of co-culture. The expression levels of key wound-healing-related genes were analyzed by qRT-PCR, as described in the “Quantitative real-time polymerase chain reaction analysis” section.

### Full-thickness skin wound model

Five-week-old female athymic mice (BALB/c-nude; Orient Bio, Seongnam, Korea) were anesthetized by intraperitoneal injection of 150 μl of ketamine (100 mg/kg) and xylazine (20 mg/kg) diluted in a normal saline solution. A square-shaped dorsal skin area (2 cm × 2 cm, 4 cm^2^) was surgically removed. After the surgery, eight 6-0 sutures (AILEE Co., Busan, Korea) were placed at the border of each wound to prevent wound collapse due to skin contracture [19]. Immediately after skin wound modeling, the mice were subdivided into 3 groups: no treatment (NT, PBS injection + Tegaderm), hMSC injection (SC group, 1 × 10^6^ cells/200 μl + Tegaderm), and ZFN-loaded hMSC injection (SC@ZFNs group, 1 × 10^6^ cells/200 μl + Tegaderm). In the cell-injected groups, hMSCs were delivered to the wound sites by subcutaneous injection at 8 sites around the wound edges (25 μl per site). All groups were treated with Tegaderm (3M Healthcare, St. Paul, MN, USA). All animals received care according to the Guidelines for the Care and Use of Laboratory Animals of Sungkyunkwan University (SKKUIACUC2020-01-12-1).

### Assessment of wound healing efficacy

The skin-covered areas of wounds were quantified by assessing photographs taken at 0, 3, 7, 10, and 14 d. The pixel sizes of areas were adjusted according to the ruler taken with and calculated using Photoshop CC (Adobe Systems). Skin-covered areas were estimated as follows: [1 − (pixel size of the wound at each time point)/(pixel size of the wound on day 0)] × 100%.

### Histology and immunohistochemistry

Mouse skin tissue specimens were retrieved 14 d posttreatment, fixed with 4% formaldehyde solution, and embedded in optimum cutting temperature compound (SciGen Scientific, Gardena, USA). Frozen samples were obtained with 10-μm sections. Sections were stained with hematoxylin and eosin to examine skin wound healing. To perform immunohistochemistry, anti-involucrin, anti-smooth muscle actin (anti-SM-α), anti-collagen 1 (Abcam), and anti-keratin 14 (anti-KRT14; BioLegend, San Diego, CA, USA) were visualized with fluorescein isothiocyanate-conjugated secondary antibodies (Jackson Immuno Research Laboratories, West Grove, PA). The sections were counterstained with 4′,6-diamidino-2-phenylindole and were examined using a fluorescence microscope (DFC 3000 G).

### qRT-PCR for in vivo specimens

For RNA extraction, mouse skin samples were collected and homogenized in TRIzol (Life Technologies, Inc., CA, USA) and chloroform. The remaining steps were the same as in the “Quantitative real-time polymerase chain reaction analysis” section. The expression of various genes (*β-actin*, *Vegf*, *Gdnf*, *Ngf*, and *S100a4*) was analyzed with qRT-PCR. The primer sequences used for qRT-PCR (in vivo) are listed in Table 2.

**Table 2.** Sequences of qRT-PCR primers (in vivo)

Primer	Sequence
Mouse *β-actin*	F: 5′-GGC TGT ATT CCC CTC CAT CG-3′ R: 5′-CCA GTT GGT AAC AAT GCC ATG T-3′
Mouse *Vegf*	F: 5′-GAG TCT GTG CTC TGG GAT TTG-3′ R: 5′-TTG GCA CGA TTT AAG AGG GGA-3′
Mouse *Gdnf*	F: 5′-TCC AGA GGG AAA GGT CGC AG-3′ R: 5′-CCT CCT TGG TTT CAT AGC CC-3′
Mouse *Ngf*	F: 5′-GGG GAG CGC ATC GAG TTT T-3′ R: 5′-CAC TGA GGT GAG CTT GGG TC-3′
Mouse *S100a4*	F: 5′-ACT TGG ACA GCA ACA GGG AC-3′ R: 5′-TTC CGG GGC TCC TTA TCT GG-3′

### Statistical analysis

Quantitative data are expressed as mean ± standard deviation. Statistical analyses were performed using GraphPad Prism (GraphPad Software, San Diego, CA, USA). Statistical significance was determined using one-way analysis of variance and unpaired Student *t* tests. Differences of *P* < 0.01 and *P* < 0.05 were considered significantly different from the control.

## Results and Discussion

### Characterization of the synthesized ZFNs

**Fig. 1.**
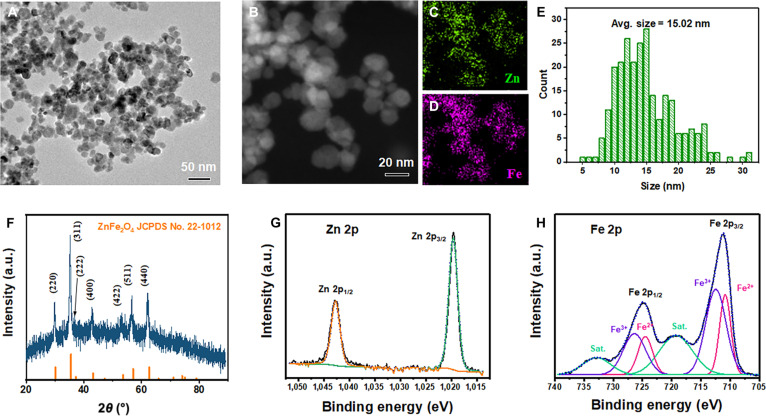
Characterization of zinc- and iron-ion-releasing nanoparticles (ZFNs). (A) The representative transmission electron microscopy (TEM) image. (B) High-angle annular dark-field imaging (HAADF) scanning transmission electron microscopy (STEM) image with (C) Zn and (D) Fe mapping data. (E) Size distribution and (F) x-ray diffraction (XRD) pattern of ZFNs. X-ray photoelectron spectroscopy (XPS) spectra of (G) Zn 2p and (H) Fe 2p.

ZFNs were synthesized by reacting Zn and Fe precursors with an appropriate concentration of NaOH in the presence of PEG as a stabilizer. The size of the spherical ZFNs synthesized at a high stirring rate of 1,000 rpm was around 15 nm (Fig. 1A to E). Energy-dispersive x-ray spectroscopy mapping analyses verified that Zn and Fe were densely and evenly distributed according to the location of the ZFNs (Fig. 1B to D). The XRD patterns of the synthesized ZFNs were well matched with the reference peaks of ZnFe_2_O_4_ (JCPDS No. 22-1012) in position and relative intensity (Fig. 1F). Since there is a limit to clearly distinguishing iron oxide-based materials only with XRD patterns, it was necessary to double-check the fact that the final product was ZFNs by figuring out the charge states of Zn and Fe (Fig. 1G and H). As a result of x-ray photoelectron spectroscopy analysis, the Zn 2p_3/2_ peak at 1,019.6 eV and the Zn 2p_1/2_ peak at 1,042.7 eV clearly indicated the presence of Zn^2+^ [20]. In the case of Fe, the Fe 2p peak could be divided into a peak at 710.78 eV for Fe^2+^ and a peak at 712.44 eV for Fe^3+^ [21,22]. Based on the higher intensity of the Fe^2+^ peak compared to that of the Fe^3+^ peak, we could conclude that the final product was ZFNs as the main product, although Fe_3_O_4_ was mixed in a small amount.

Compared to ZFNs synthesized at a low stirring rate of 400 rpm (Fig. S1), the size and shape uniformity of the NPs were noticeably improved (Fig. 1A to E). NPs are generally known to have much higher reactivity because their specific surface area increases exponentially compared to that of the bulk state, and due to this advantage, they have been used as promising materials for various applications related to reactivity [23,24]. However, since not all applications require high responsiveness unconditionally, a sophisticated adjustment process was required to reach the set target value. In this regard, controlling the size of the NPs was a key factor, since the surface area adjusted according to the size of the NPs is closely related to the reaction rate in a positive direction. As an example, Morais et al. [25] reported the results of gradually decreasing the diameter of neodymium-doped yttrium aluminum garnet NPs in the direction of gradually increasing stirring speed. In addition, Prabu et al. [26] reported the results of reducing the size of nanosilica by increasing the stirring speed. As such, stirring speed is one of the efficient parameters that can most easily control the size of NPs, and usually, the faster the stirring speed, the smaller the particles that can be obtained as the final product. This is because the shear forces operating at higher speeds are increased, cracking the particles to smaller diameters [27]. Furthermore, when the particle size is small, particles of a more uniform size can generally be observed, and uniform size is an important factor that makes the performance of NPs constant. NPs of nonuniform size can cause deviations in reactivity and ion dissolution rate according to size even within one system, and this is likely to be an unclear factor in the process of verifying and investigating the performance of NPs. Going one step further, nonuniform NP sizes negatively affect the reproducibility of catalyst synthesis and utilization.

### Ion release kinetics and cytotoxicity assessment based on ZFNs

**Fig. 2.**
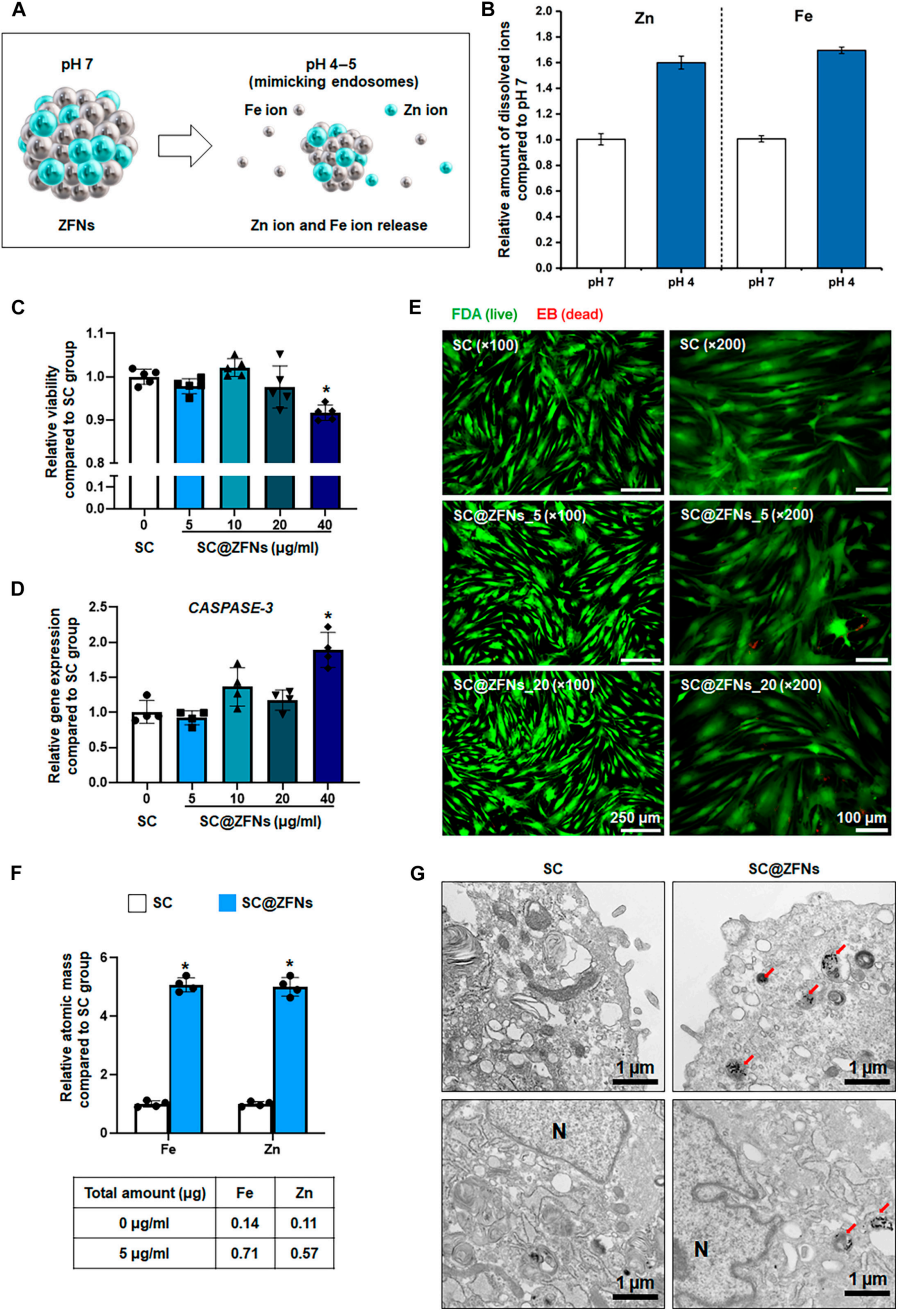
pH-responsive dual-ion release and biocompatibility of ZFNs. (A) Schematic diagram depicting Zn and Fe ion release under low-pH conditions. (B) Cumulative dissolution of Zn and Fe ions at different pH values. (C) Cell viability of human mesenchymal stem cells (hMSCs) treated with ZFNs at various concentrations, measured by the Cell Counting Kit-8 (CCK-8) assay (*n* = 5, *P < 0.05 vs. SC group). (D) Relative expression of *CASPASE-3* in hMSCs after 24-h ZFN treatment (*n* = 4, *P < 0.05 vs. SC group). (E) Fluorescein diacetate (FDA)/ethidium bromide (EB) staining of hMSCs after ZFN treatment (live cells: green; dead cells: red). (F) Intracellular Zn and Fe levels in SC@ZFNs compared to those in SC, measured by inductively coupled plasma mass spectrometry (ICP-MS) (*n* = 4, *P < 0.05 vs. SC group). (G) TEM images of hMSCs after 24 h of treatment with ZFNs. Red arrows indicate intracellular ZFNs, and N denotes the nucleus.

We aimed to assess whether Zn and Fe ions could be effectively released from ZFNs after moving inside the cells without inducing cytotoxicity. Considering our NPs’ size (~15 nm), ZFNs are expected to be internalized via endocytosis and subsequently localized within endosomal compartments, where the mildly acidic environment can facilitate ion release. To evaluate the released Zn and Fe ions within cellular environments, the ZFNs were incubated in pH 4 and 7 solutions, mimicking endosome conditions, respectively. We then measured the amount of ion elution over time (Fig. 2A and B and Fig. S2A). The increase ratios in Zn and Fe eluted at 24 h (cumulative elution increased at 24 h compared to that at 4 h) were 12.14% for Zn and 12.56% for Fe at pH 7 but 52.19% for Zn and 52.25% Fe for at pH 4. These observations demonstrate that Zn and Fe could be effectively released from ZFNs after trapping in endosomes.

Subsequently, we investigated the biocompatibility of the synthesized NPs. In this study, SC refers to untreated hMSCs, while SC@ZFNs denote hMSCs treated with ZFNs for 24 h. We found that ZFNs did not affect cell viability at concentrations up to 20 μg/ml (Fig. 2C). Gene expression analysis revealed no significant changes in the expression of *CASPASE-3*, an indicator of apoptotic cell death, at these concentrations (Fig. 2D). Validation using FDA/EB staining confirmed the absence of cell apoptosis at ZFN concentrations of 5 and 20 μg/ml (Fig. 2E). For all subsequent experiments, SC@ZFNs were generated by treating hMSCs with 5 μg/ml of ZFNs. Although concentrations up to 20 μg/ml did not induce cytotoxicity, 5 μg/ml was selected as the minimal effective dose to ensure sufficient ion release while minimizing potential cellular stress and maintaining optimal conditions for long-term culture and paracrine activity. Further tests were conducted to measure the intracellular ion concentrations after treating cells with 5 μg/ml of ZFNs for 24 h (Fig. 2F). Results showed that cells treated with ZFNs contained significantly higher amounts of Zn and Fe ions compared to untreated cells. These results confirmed that ZFNs could successfully increase the intracellular concentration of Zn and Fe ions without inducing cellular damage. To confirm the intracellular localization of ZFNs, transmission electron microscopy imaging was performed after treating hMSCs with ZFNs for 24 h (Fig. 2G). NPs were observed within intracellular vesicles with morphologies consistent with those of endosomes and lysosomes [28,29]. These finding suggest that ZFNs were internalized into the cells through endocytosis [30]. The ability of ZFNs to preferentially release dual ions at low pH levels underscores their potential for targeted therapeutic applications. Notably, when ZnO and Fe_3_O_4_ NPs were administered either separately or in combination, only the mixture group exhibited a detectable decrease in cell viability (Fig. S2B). In contrast, ZFNs showed no such cytotoxicity despite releasing a greater amount of Zn^2+^ and Fe^3+^ ions under acidic conditions (Fig. S2A). This can be attributed to the relatively low crystallinity and spinel structure of ZFNs, which facilitate controlled ion release rather than uncontrolled accumulation [31,32]. Such a release pattern is likely to reduce the persistence of residual particles in cells and thereby minimize cytotoxicity. Taken together, these findings indicate that ZFNs uniquely combine efficient dual-ion release with high biocompatibility, underscoring their potential advantage over conventional single-metal oxide NPs in stem-cell-based therapies.

### Effect of ion release from ZFNs to hMSCs

**Fig. 3.**
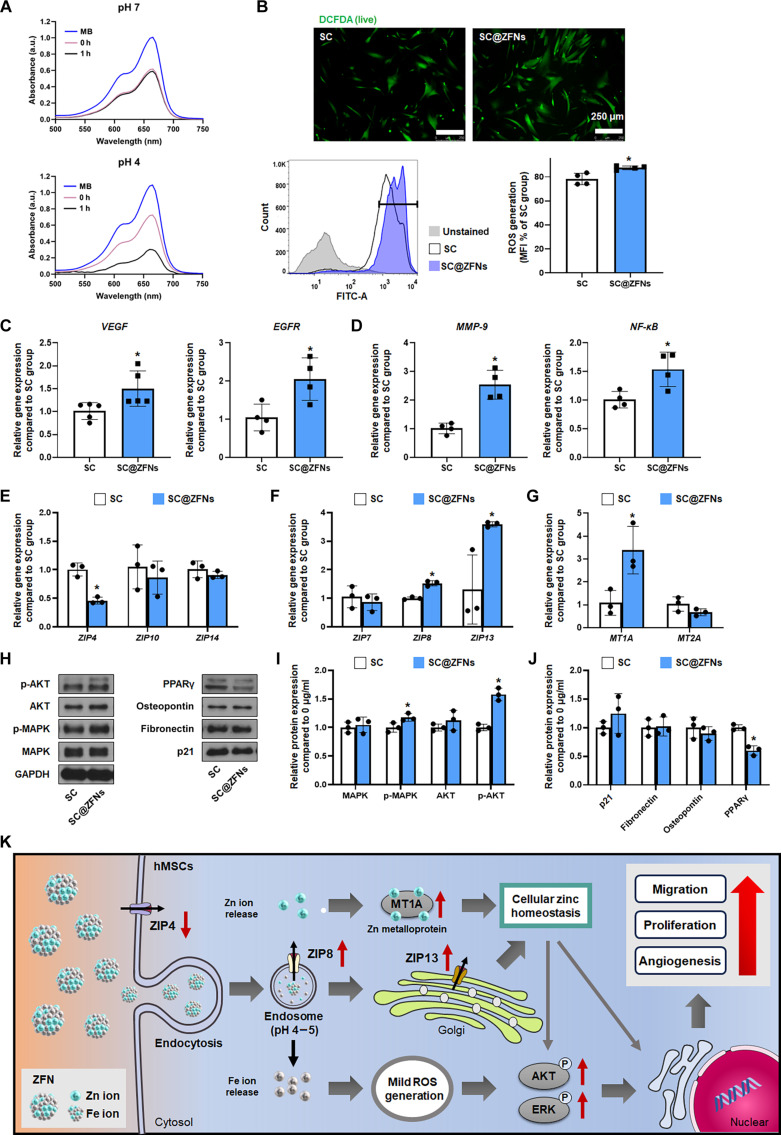
The effect of Zn and Fe ions released from ZFNs on the wound healing efficacy of hMSCs. (A) Methylene blue (MB) assay for detecting pH-dependent reactive oxygen species (ROS) generation by ZFNs. (B) Intracellular ROS staining with dichlorodihydrofluorescein diacetate (DCFDA; green) and its quantification (*n* = 4, *P < 0.05 vs. SC group). Relative expression of (C) angiogenesis- and (D) migration-related genes in hMSCs after 24-h ZFN treatment (*n* = 4, *P < 0.05 vs. SC group). Relative expression of (E) zinc transporters located on the cell membrane, (F) zinc transporters located within the cell, and (G) zinc-ion-storage-related genes in hMSCs after 24-h ZFN treatment (*n* = 4, *P < 0.05 vs. SC group). (H to J) Protein expression level in hMSCs after 24-h ZFN treatment and its quantification (*n* = 3, *P < 0.05 vs. SC group). (K) Schematic illustration describing the effect of Zn and Fe ions on hMSCs. FITC, fluorescein isothiocyanate; MFI, mean fluorescence intensity; MAPK, mitogen-activated protein kinase.

To investigate the effects of ZFN-induced ion release on hMSCs, we examined the changes in cellular behavior attributed to increased intracellular concentrations of Zn and Fe ions. Fe ions are known to cause mild ROS generation within the cells. To assess whether the ROS observed in our system could be linked to the Fenton-like activity of released Fe ions, an absorbance-based MB assay was conducted. MB absorbance remained unchanged at pH 7 but gradually decreased under acidic conditions (pH 4), indicating ROS generation in environments mimicking endosomes (Fig. 3A). This suggests that ZFNs may induce mild ROS production through Fe ion release under low-pH conditions. Our results demonstrated that hMSCs treated with ZFNs showed a significant increase in ROS levels compared to the untreated control (Fig. 3B). This increase in ROS was associated with heightened expression of vascular endothelial growth factor (*VEGF*) and epidermal growth factor receptor (*EGFR*), both critical for angiogenesis, in the SC@ZFNs group (Fig. 3C). Additionally, we observed an increase in the expression of matrix metalloproteinase-9 (*MMP-9*), related to cell migration, and nuclear factor kappa-light-chain-enhancer of activated B cells (*NF-κB*), associated with anti-apoptotic responses, in the SC@ZFNs group (Fig. 3D). Given that both MMP-9 and NF-κB are redox-sensitive factors, their upregulation in the SC@ZFNs group is likely a response to the moderate increase in ROS induced by Zn and Fe ion release. The upregulation of NF-κB, a key regulator of immune response and cell survival, further indicates a strengthening of the cellular defense mechanisms, promoting tissue repair and regeneration in wound healing contexts [33].

The impact of zinc ion release influenced various zinc channels and transporters. Notably, the expression of *ZIP4*, a zinc transporter responsible for zinc uptake, decreased significantly in the SC@ZFNs group (Fig. 3E). ZIP4 is primarily localized on the plasma membrane, where it facilitates the import of extracellular zinc into the cell [34]. Conversely, expressions of ZIP8 and ZIP13, which help in regulating intracellular zinc levels, were elevated in the SC@ZFNs group (Fig. 3F). ZIP8 is predominantly located on the plasma membrane, where it transports zinc into the cytosol. ZIP13, in contrast, is localized to intracellular organelles, such as the Golgi apparatus and endoplasmic reticulum, where it modulates zinc distribution within cellular compartments, thus playing a key role in intracellular signaling and homeostasis [35]. Moreover, the expression of metallothionein 1A (*MT1A*), which stores Zn ions, was also increased in the SC@ZFNs group (Fig. 3G). These changes suggest a robust modulation of cellular zinc homeostasis. Considering that Fe ions released from SC@ZFNs can induce moderate oxidative stress, the upregulation of MT1A may also reflect a protective cellular response to ROS, contributing to enhanced antioxidant capacity and cellular resilience under stress conditions [36]. The observed increase in zinc transporters and metallothionein in the SC@ZFNs group suggests a protective cellular response against zinc-induced cytotoxicity, which is crucial for maintaining cell viability under stress conditions induced by zinc overload. Enhanced zinc regulation also supports the activation of repair pathways and inflammatory responses, which are vital for effective wound healing. Further analysis revealed that the presence of Zn and Fe ions led to the activation of critical cell signaling pathways, specifically AKT and mitogen-activated protein kinase (MAPK) signaling (Fig. 3H and I). These pathways are pivotal in promoting cell survival, proliferation, and differentiation. However, there was no significant change in the expression of senescence or differentiation markers, such as PPARγ, which in fact showed a decrease in expression (Fig. 3H and J). The intracellular delivery of Zn and Fe ions via ZFNs critically influenced hMSC responses. The generation of mild ROS by iron ions, coupled with the management of cellular zinc homeostasis by zinc ions, activated AKT and MAPK signaling pathways. These activations contributed to the increased expression of factors involved in angiogenesis, migration, and proliferation (Fig. 3K) [37]. Collectively, these findings illustrate that ZFNs can modulate stem cell functionality through ion release, markedly enhancing their regenerative potential.

### Therapeutic effect of SC@ZFNs in fibroblasts and keratinocytes

**Fig. 4.**
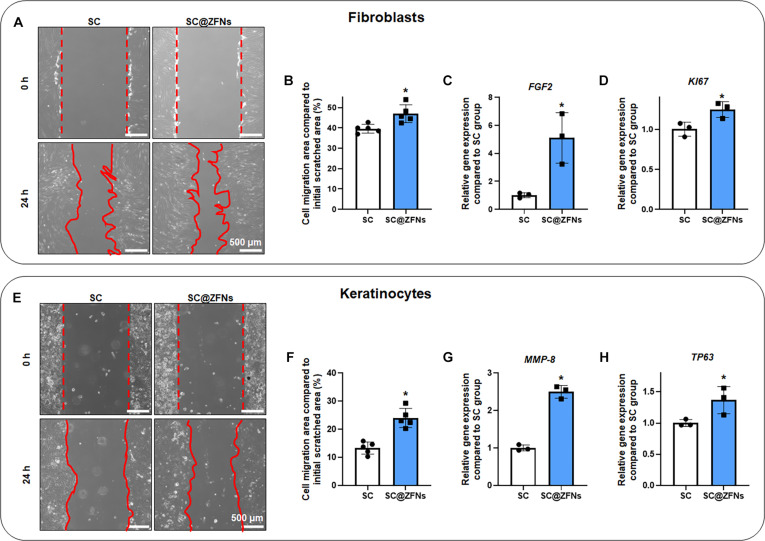
Enhanced wound healing ability of SC@ZFNs. (A and B) Scratch assay of fibroblasts co-cultured with the SC@ZFNs group for 24 h and quantification of cell migration (*n* = 5, *P < 0.05 vs. SC group). Relative expression of (C) *FGF2* and (D) *KI67* in fibroblasts after 24-h co-culture with the SC@ZFNs group (*n* = 3, *P < 0.05 vs. SC group). (E and F) Scratch assay of keratinocytes co-cultured with the SC@ZFNs group for 24 h and quantification of cell migration (*n* = 5, *P < 0.05 vs. SC group). Relative expression of (G) *MMP-8* and (H) *TP63* in keratinocytes after 24-h co-culture with the SC@ZFNs group (*n* = 3, *P < 0.05 vs. SC group).

To investigate the enhanced therapeutic efficacy of SC@ZFNs, we explored the paracrine factor secreted by SC@ZFNs and their effects on skin cells, specifically fibroblasts and keratinocytes. We utilized a transwell co-culture system to culture SC@ZFNs with fibroblasts and keratinocytes for 24 h. The system allowed us to assess the effect of the NP-enhanced paracrine factor secretion from hMSCs on skin cells. The co-culture of fibroblasts with the SC@ZFNs group resulted in a notable increase in the wound healing ratio of fibroblasts (Fig. 4A and B). This improvement was accompanied by upregulation of *FGF2* and *KI67*, both of which are crucial for the proliferation phase of wound healing (Fig. 4C and D). Similarly, the SC@ZFNs group exhibited an increased wound healing ratio of keratinocytes (Fig. 4E and F). There was a significant rise in the expression of *MMP-8*, which is associated with the cell migration and remodeling phases of wound healing (Fig. 4G). Additionally, the keratinocyte proliferation marker *TP63* showed increased expression (Fig. 4H). The application of SC@ZFNs significantly enhanced the migration and proliferation of both fibroblasts and keratinocytes. In fibroblasts, the increased expression of *FGF2* and *KI67* induced by SC@ZFNs suggests a robust therapeutic potential during the proliferation phase of wound healing [38,39]. In keratinocytes, the upregulated expression of *MMP-8* may contribute to improving remodeling capabilities, while the increased *TP63* expression suggests a boost in the proliferation stage [40,41]. These findings highlight the potential of NP-enhanced stem cells to mediate effective paracrine interactions, substantially improving wound healing dynamics. To further investigate this, we analyzed the secretion of key paracrine signaling molecules involved in wound repair. Enzyme-linked immunosorbent assay results revealed that the SC@ZFNs group showed a significant increase in the secretion of VEGF and KGF compared to the SC group (Fig. S3). VEGF plays a role in recruiting fibroblasts to the wound site and facilitating their activation, contributing to granulation tissue formation and enhancing the conditions for epithelial cell migration [42]. KGF (also known as FGF7) primarily acts on keratinocytes, encouraging their proliferation and differentiation. This activity supports the re-epithelialization process and plays an essential role in restoring the integrity of the skin barrier [43]. These enhanced paracrine factors contribute to the improved healing capabilities observed in the co-cultured skin cells. By supporting the activity of both fibroblasts and keratinocytes, SC@ZFNs may contribute to a more coordinated and efficient healing process.

### Therapeutic efficacy of SC@ZFNs in wound healing

**Fig. 5.**
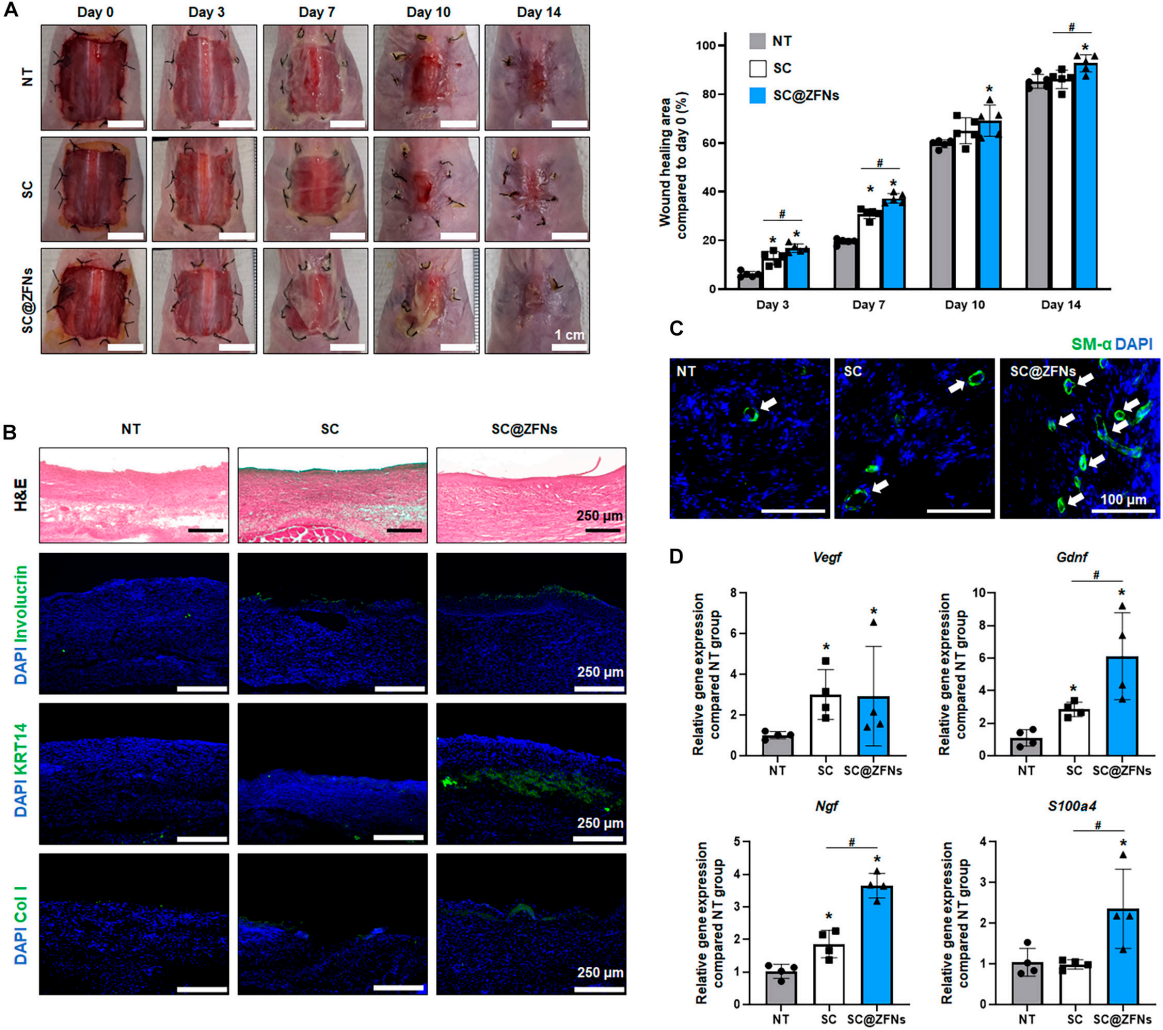
In vivo wound closure induced by hMSCs treated with ZFNs. (A) Representative images of wound closure at 0, 3, 7, 10, and 14 d and quantification in all groups (*n* = 5, *P < 0.05 vs. no treatment [NT] group, ^#^*P* < 0.05 vs. each group). (B) Hematoxylin and eosin (H&E) staining and immunostaining of wound tissue at day 14 for involucrin (green), KRT14 (green), and collagen type I (Col I; green). (C) Immunostaining for smooth muscle actin (SM-α; green) with 4′,6-diamidino-2-phenylindole (DAPI; blue) in the skin wound site at 14 d after the treatments. White arrows indicate SM-α. (D) Relative expression of *Vegf*, *Gdnf*, *Ngf*, and *S100a4* in the wound region at 14 d after the treatments (*n* = 4, *P < 0.05 vs. NT group, ^#^*P* < 0.05 vs. each group).

The therapeutic efficacy of SC@ZFNs was assessed in an in vivo mouse model to determine their potential in enhancing wound healing. Over a 14-d observation period, it was evident that the wounds treated with SC@ZFNs healed significantly faster compared to other groups (Fig. 5A). Notably, starting from day 3 posttreatment, the SC@ZFNs group demonstrated accelerated wound healing, a trend that persisted until day 7. By day 14, this group exhibited the most pronounced wound healing among all groups. Histological examination of the tissue at day 14 revealed that the areas treated with SC@ZFNs showed superior healing (Fig. 5B and C). Importantly, the expression levels of skin-related proteins such as involucrin, KRT14, and collagen type I (Col I) on wound sites were markedly higher in the SC@ZFNs group compared to those in the other groups (Fig. 5B). Involucrin, a key component of the epidermal barrier, reflects the formation of a stratified epithelium [44]. KRT14 serves as a basal keratinocyte marker associated with epidermal regeneration, while Col I indicates extracellular matrix remodeling within the dermal layer [45,46]. Together, the upregulation of these markers suggests coordinated enhancement of both barrier restoration and structural tissue repair. Additionally, there was a noticeable increase in vascular distribution within the healed tissues of the SC@ZFNs group, indicating enhanced angiogenesis (Fig. 5C). The expression of SM-α, indicative of improved tissue remodeling and maturation, was mainly localized in the dermal layer, corresponding to myofibroblasts beneath the epidermis [47,48]. Consistent with protein expression results, the gene expression also reflected the enhancement to regenerative activity. The gene expression analysis correlated with the observed improvements in tissue structure and composition (Fig. 5D). The expression of *Vegf*, *Gdnf*, *Ngf*, and *S100a4* were improved in the SC@ZFNs group. This enhancement suggests that SC@ZFNs foster a conducive environment for vascularization, which is crucial for nutrient and oxygen supply to the regenerating tissues, and nerve integration, which is essential for restoring functionality and sensitivity to damaged tissues. The upregulation of these factors thereby indicates a comprehensive approach to tissue repair, enhancing both the structural and functional aspects of healing.

The transplantation of SC@ZFNs into skin lesions markedly accelerated and enhanced the therapeutic efficacy of acute wound closure and re-epithelialization. In the histological analysis on day 14, no immunogenic responses or other unintended side effects were observed. This observation is in line with previous studies utilizing ion-releasing NPs, particularly those based on zinc or iron, which have consistently reported minimal long-term toxicity and favorable in vivo biocompatibility in regenerative applications [49,50]. The NPs used in this study were also presumed to be fully ionized after cellular uptake, with no residual particles remaining in the tissue. Therefore, the potential for particle-associated toxicity or long-term adverse effects is expected to be considerably lower compared to that of conventional NP systems. These findings demonstrate the profound impact of integrating ZFNs with stem cell therapy, offering a promising approach for improving the outcomes of wound closure in clinical settings. This constructive interaction between ZFNs and stem cells not only speeds up the healing process but also ensures the restoration of skin functionality and appearance, making it a valuable strategy for managing acute skin injuries.

## Conclusion

The integration of ZFNs with hMSCs marks a notable advancement in the field of regenerative medicine, particularly in enhancing wound healing processes. Our study demonstrated that ZFNs, designed to be pH sensitive, effectively modulate the intracellular environment of hMSCs by regulating crucial zinc transporter genes and enhancing the expression of genes involved in angiogenesis and cellular migration. This was accompanied by the activation of MAPK and AKT signaling pathways, highlighting the intricate cellular mechanisms influenced by ZFNs. Furthermore, hMSCs treated with ZFNs not only showed improved cellular responses but also enhanced the migratory capacity and tissue regeneration efficacy in fibroblasts and keratinocytes. In vivo experiments provided compelling evidence of the efficacy of this approach, with SC@ZFNs markedly accelerating wound healing and promoting the expression of critical skin barrier proteins in mouse models. Although our data reveal strong correlations between ROS modulation, zinc transporter regulation, and enhanced wound healing outcomes, we acknowledge that further work will be required to directly validate the causal contribution of these pathways. Nevertheless, the consistency of our findings across multiple in vitro and in vivo models, together with supporting evidence from prior reports, provides a strong biological rationale for this mechanism.

These outcomes suggest that SC@ZFNs may contribute to improved wound healing dynamics by enhancing cellular coordination and supporting more effective tissue repair. Overall, the application of ZFNs in stem cell therapies presents a promising approach for enhancing wound healing outcomes. However, to fully realize the clinical potential of this approach, further development of methodologies enabling precise, sequential, or selective release of multiple therapeutic ions is required. This will allow broader and more effective application across various disease models. By enhancing the intrinsic regenerative capabilities of hMSCs and promoting beneficial interactions among skin cells, ZFNs offer a promising avenue for improving outcomes in wound management and tissue repair. This study not only underscores the potential of integrating NP technology with biological therapies but also sets the stage for future clinical applications where accelerated and enhanced healing is critical.

## Data Availability

Data will be made available on request.
