# Supplementary material for: Synergistic Ion-Releasing Nanoparticles as a Therapeutic Platform for Modulating Adult Stem Cell Activity in Wound Healing
Source: Biomater Res. 2025 Dec 3;29:0281. doi: 10.34133/bmr.0281 (PMC12673018; doi:10.34133/bmr.0281)
Supplement: Supplementary 1 — Figs. S1 to S3 [file bmr.0281.f1.docx]

**Synergistic ion-releasing nanoparticles as a therapeutic platform for modulating adult stem cell activity in wound healing**

Yu-Jin Kim^1,2,†^, Jaeyoung Lee^3,4,†^, Eun-Cheol Lee^1,†^, Jiwoo Song^3^, Yonghwan Jo^3^, Han Young Kim^5^, Taekyung Yu^3,^*, and Suk Ho Bhang^1,^*

^1^ School of Chemical Engineering, Sungkyunkwan University, Suwon 16419, Republic of Korea

^2^ Center for Biomaterials Research Center, Korea Institute of Science and Technology, Seoul, 02792, Republic of Korea

^3^ BK21 FOUR Integrated Engineering Program, Department of Chemical Engineering, Kyung Hee University, Yongin 17104, Republic of Korea

^4^ School of Earth and Atmospheric Sciences, Georgia Institute of Technology, Atlanta, GA 30332, USA

^5^ Department of Biomedical-Chemical Engineering, The Catholic University of Korea, Bucheon, 14662, Gyeonggi, Republic of Korea

^†^These authors contributed equally to this work

*Co-corresponding authors

E-mail: tkyu@khu.ac.kr (T.K.Y.), sukhobhang@skku.edu (S.H.B.)


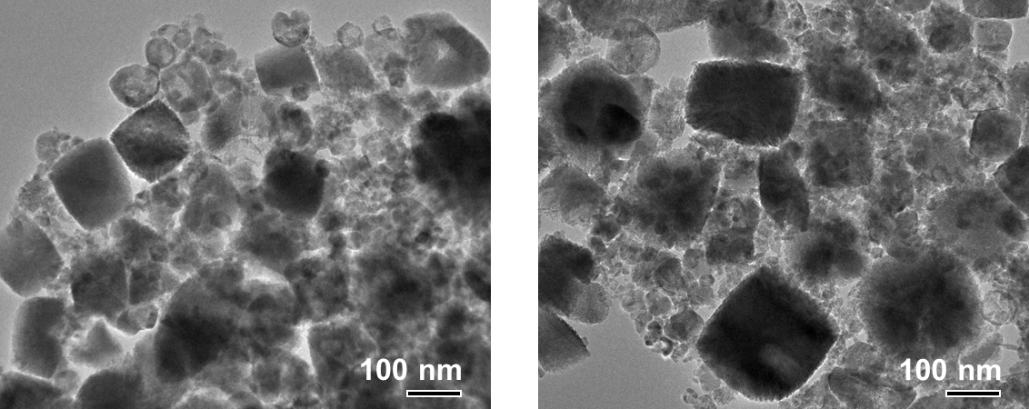


**Fig S1.** Representative TEM images of ZnFe_2_O_4_ NPs synthesized at 400 rpm condition.


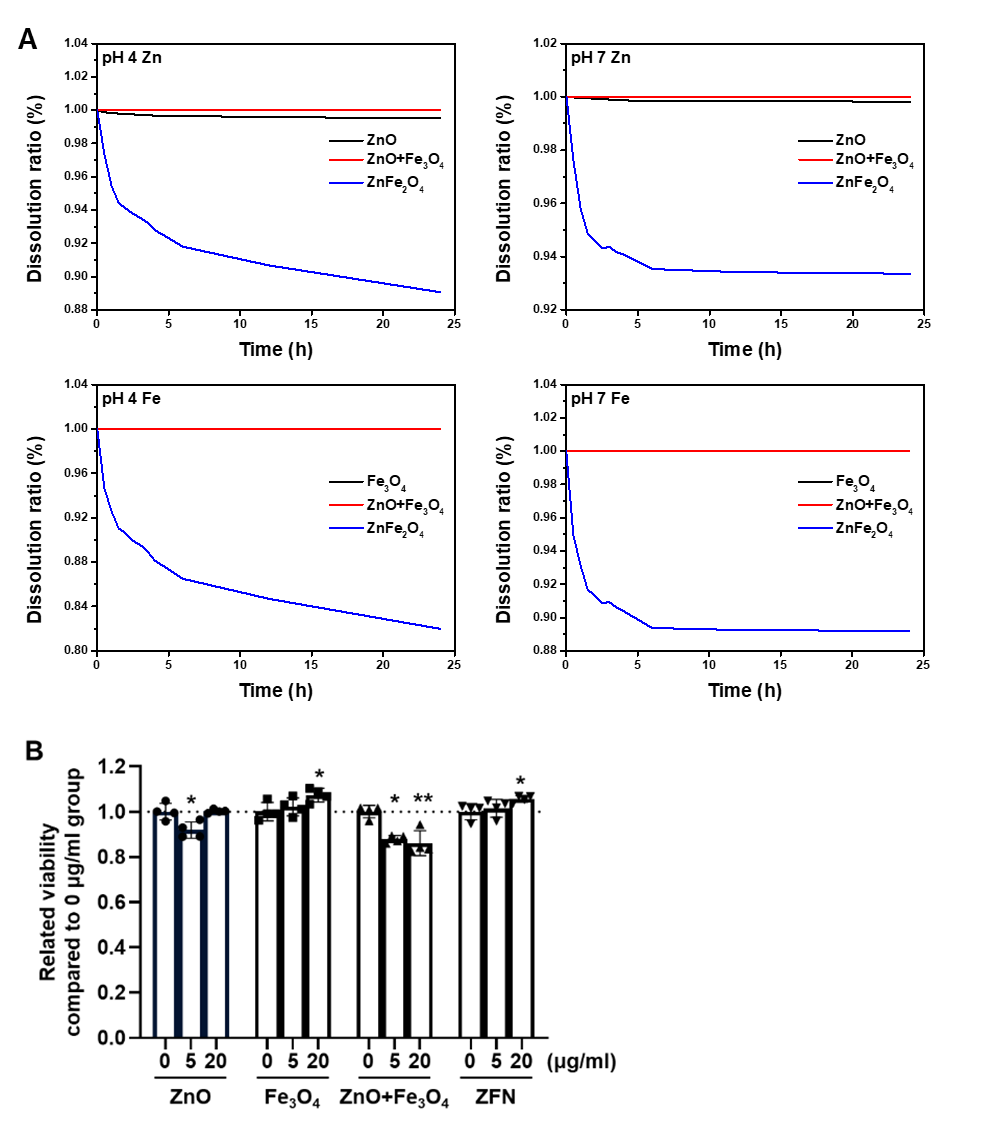


**Fig S2.** Ion release kinetics and cytotoxicity assessment of ZFNs. (A) Ion dissolution profile. (B) The relative cell viability ratio was evaluated by CCK-8 using the 0 μg/ml group (no treatment cells) as a control (n = 4, *p < 0.05 vs. 0 μg/ml group).

**
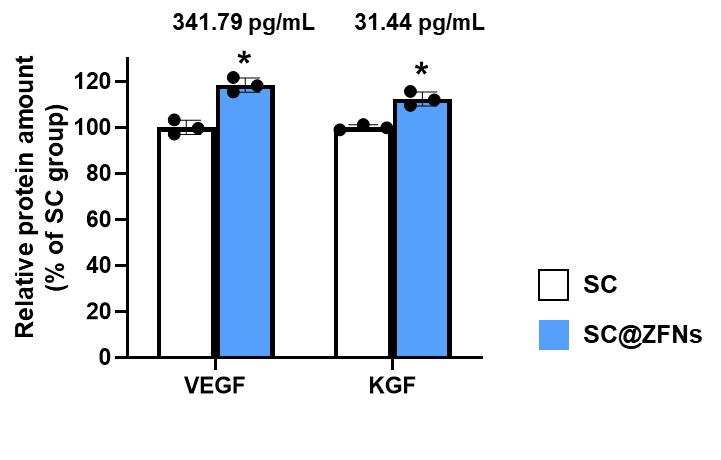
**

**Fig S3.** Concentration of VEGF and KGF in the CM derived from SC or SC@ZFNs groups, as evaluated by ELISA (n = 3, *p < 0.05 vs SC group).
